# Bonding Strength of the CFRP and AA6061 Joint Using Ascorbic Acid and Sodium Chloride Surface Treatment

**DOI:** 10.3390/ma19122594

**Published:** 2026-06-16

**Authors:** Donggil Kang, Jaeha Kim, Hogyeong Seong, Jaejun Yoon, Seungboo Jung

**Affiliations:** 1 Department of Advanced Materials Science & Engineering, Sungkyunkwan University, 2066 Seobu-ro, Jangan-gu, Suwon 16419, Republic of Korea; 2 Department of Semiconductor Convergence Engineering, Sungkyunkwan University, 2066 Seobu-ro, Jangan-gu, Suwon 16419, Republic of Korea

**Keywords:** adhesive bonding, heterogeneous bonding, surface treatment, surface free energy, bonding strength

## Abstract

The adhesive bonding of aluminum with other materials is widely used in the aerospace, marine, automotive and railroad industries that require lightweight materials. Adhesive bonding has the advantages of reduced corrosion, stress concentration, and cost effectiveness. To improve bonding strength and performance, we examined the use of ascorbic acid (vitamin C), which is a water-soluble compound and a natural reducing agent. Owing to its reducing power and acidity, ascorbic acid allows the Al etching process to proceed efficiently to increase the surface roughness and prevent Al oxidation. In addition, this study used an eco-friendly technique of simply immersing aluminum substrates in an ascorbic acid solution with sodium chloride. The surface free energy was evaluated using the sessile drop method and calculated using the Owens–Wendt–Rabel and Kaelble method. Confocal microscope was used to investigate the roughness of the surface, and the functional groups of Al surface were analyzed by X-ray photoelectron spectroscopy. The bonding strength was measured using the single-lap joint shear test. Compared to aluminum without treatment, the bonding strength of a treated AA 6061 was enhanced by 58.6%.

## 1. Introduction

In recent years, the aerospace, ocean, vehicle, and railroad industries have required more lightweight materials. This demand has increased the use of lighter alloys, polymers, and composites to reduce weight. However, lightweight materials are used in combination with other materials; thus, dissimilar joining methods are required [1,2,3,4,5]. A conventional method using bolts or rivets provides superb joining strength; thus, it is widely used in engineering. On the other hand, conventional methods result in increased weight, low sealing capacity, decreased structural area (owing to bolt holes), increased stress, and damage to structures, which has disadvantages for joints [5,6].

Given this recent trend, adhesive bonding is one of the optimal methods. Adhesive bonding allows for lightweight structure because bolts and rivets are not required, which prevents structural weakening, stress concentration, fatigue phenomena, and resistance reduction [5,6,7]. Adhesive bonding is a straightforward method for joining dissimilar materials at room temperature and without environmental restrictions. Thus, adhesive bonding is more efficient in terms of designing structures and materials selection than other joining methods. In addition, adhesive bonding seals the joints, avoiding galvanic corrosion [6,8], and adhesive bonding is the only available method for achieving structures involving the joining of thin-walled elements, even when those elements have different thicknesses [5,9].

In adhesive bonding, adhesion occurs by the penetration of adhesive into the irregularities of the substrate surface, which simultaneously forces out trapped air from the cavities and pores [10]. Therefore, to improve the bonding properties and performance, the roughness of the metal surface and mechanical interlocking should be increased, and the oxide layer should be removed by a pre-treatment with physical (e.g., sanding, grinding, and abrasive blasting) or chemical methods [10,11].

However, conventional chemical methods, such as using strong acids (e.g., HCl, H_2_SO_4_, HNO_3_, H_3_PO_4_) or strong bases can often severely deform the metal surface. Thus, the use of strong chemical compounds decreases the joining strength, damages the environment, and has high wastewater treatment costs [10,11]. Therefore, eco-friendly pickling solutions are preferable and required. In this study, AA 6061 was joined with a carbon fiber-reinforced plastic (CFRP), which is lightweight and has a high strength-to-weight ratio. In addition, a simple method using ascorbic acid (a water-soluble compound and a natural reducing agent) and NaCl was used for the AA 6061 etching process. NaCl was used because the corrosion rate of acids in the presence of NaCl was much higher than when only acids were used [12].

## 2. Materials and Methods

### 2.1. Materials

Based on the ASTM D5868-01 standard [13], the bonded materials, including the metal and the CFRP, were fabricated with the dimensions of 25.4 mm × 100 mm, and 2 mm thickness. The polymer substrate CFRP (CFRP, Hankuk Carbon Co., Ltd., Miryang, Republic of Korea) was manufactured using a twill weave and a unidirectional pre-preg consisting of fiber and epoxy resin. The commercial AA 6061-T6 aluminum alloy (AA 6061-T6, Specsico Corporation, Incheon, Republic of Korea) used in this work is a precipitation-hardened alloy mainly composed of aluminum (95.9–98.6%), magnesium (0.8–1.2%), and silicon (0.4–0.8%), with minor amounts of iron (≤0.7%), copper (0.15–0.4%), chromium (0.04–0.35%), and zinc (≤0.25%). In addition, a commercial adhesive (WM 5005, Won Chemical, Hwaseong, Republic of Korea), consisting of 70% bisphenol-A, 10% epoxy acrylate, and less than 1% ethylene glycol monomethyl ether acetate, together with an aliphatic amine curing agent, was employed in this study.

### 2.2. Experimental Methods

**Figure 1:**
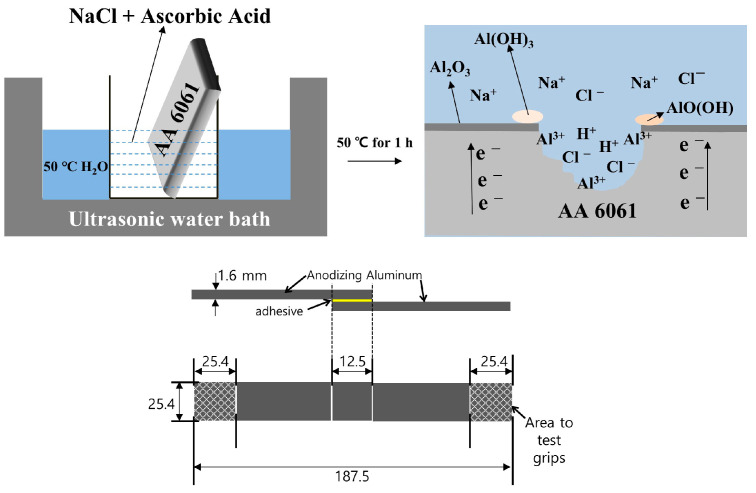
Schematic diagram of redox reaction of AA 6061 alloys with ascorbic acid solution and lap shear test specimen.

Figure 1 illustrates the overall experimental method and the lap-shear joint configuration. The aluminum substrate was immersed for 1 h in a 1 M ascorbic acid solution with different NaCl concentrations (i.e., 0.25, 0.5, and 1 M). Then, the surface of the immersed substrate was twice cleaned with DI-water. The surface free energy was evaluated by the sessile drop method and calculated by the Owens–Wendt–Rabel and Kaelble (OWRK) method. Surface roughness was measured by a confocal microscope, and functional groups of the Al surface were analyzed by X-ray photoelectron spectroscopy (K-Alpha, Thermofisher scientific, Waltham, MA, USA). The surface roughness of AA 6061 aluminum was characterized after etching in an ascorbic acid solution containing NaCl, using a 3-dimensional surface profiler (3D LSM, Olympus, Tokyo, Japan).

The adhesive was an epoxy-amine system, which is a recommended adhesive for automotive body bonding; it is a room-temperature-curing adhesive. The overlap area of the lap shear specimens where adhesive was applied, was 25.4 × 12.7 mm^2^, followed by ASTM D5868-01. The curing conditions were 25 °C and 24 h. The cut substrates were cleaned by ultrasonication with ethanol for 10 min to remove contaminants such as oil, mold agents, and dust. After curing the adhesive-bonded joints, their bonding strength was measured using a tensile test machine (Universal testing machine, R&B, Daejeon, Republic of Korea). The lap shear test was performed with a loading speed of 13 mm/min (ASTM D5868-01). The single-lap joint evaluation was performed on ten specimens for each treatment condition.

## 3. Results

**Figure 2:**
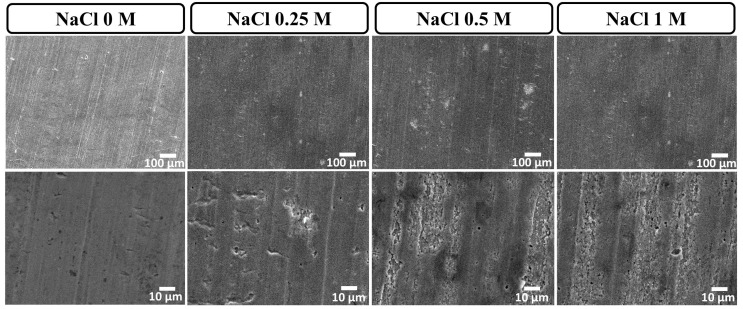
Cross-sectional micrographs of AA 6061 surface with various NaCl concentrations.

Figure 2 shows SEM micrographs of a reaction between ascorbic acid and Al that occurs during the ultrasonic immersion of the Al substrate in an ascorbic acid and NaCl solution. In the case of AA 6061, in the aqueous solution, the following reaction occurs:

$$2Al+{3H}_{2}O={Al}_{2}O_{3}+6H^{+}+6e^{-}$$

$${6H}^{+}+6e^{-}={3H}_{2}$$

$${Al}_{2}O_{3}+H_{2}O\to {Al}_{2}O_{3}\cdot nH_{2}O\to Al{\left(OH\right)}_{2}$$

and the corrosion rate of 6061 increases in the order of the neutral NaCl solution, acidic solution, acid and NaCl mixed solution, and this result can be confirmed through a SEM micrograph. In the ascorbic acid aqueous solution, Al_2_O_3_ (which is the oxide layer of AA 6061) is removed by carboxylic acid, and Na^+^ and Cl^−^ are added to act as electrolytes and cause polarization. Due to this, Al is oxidized and loses electrons, and the generated electrons move to the Al surface to form AlOOH_3_ and Al(OH)_3_ salts. In addition, the Cl^−^ ions are adsorbed onto the surface of the AA 6061 in the aqueous solution, which attack the passivation layer of AA 6061, resulting in the disruption of the passivation layer causing pitting corrosion. The Cl^−^ ion has a strong penetrating power, which can produce tiny pores of passivation layers around the precipitation and grain boundary. In ascorbic acid aqueous solution, the interaction between the Cl^-^ ions and Al prompts an increase in corrosion rate and produces water-soluble compounds. These oxidation-reduction processes form craters on the Al surface in acidic solutions. The etching process eliminates contamination and increases surface roughness. In the case of 1 M NaCl aqueous solution, it shows that a more cratered surface is produced compared to scratches and pits observed on the untreated surface. An increase in NaCl concentration resulted in a surface with more craters and greater roughness than that at a lower NaCl concentration. When 1 M NaCl was used, the deepest and most numerous craters formed on the surface. These microstructural investigations showed that more craters were created on the surface as the concentration of NaCl increased because more anions that could form different complexes with Al were present. In addition, NaCl acted as an electrolyte, thus, polarization occurred by transferring electrons from Al^3+^ ions to the solution, and Cl^–^ ions promoted the dissolution of an Al oxide layer in the acidic solution [14]. These created craters removed contaminants from the surface through the etching process and increased the roughness of the surface, thereby increasing the mechanical interlocking.

**Figure 3:**
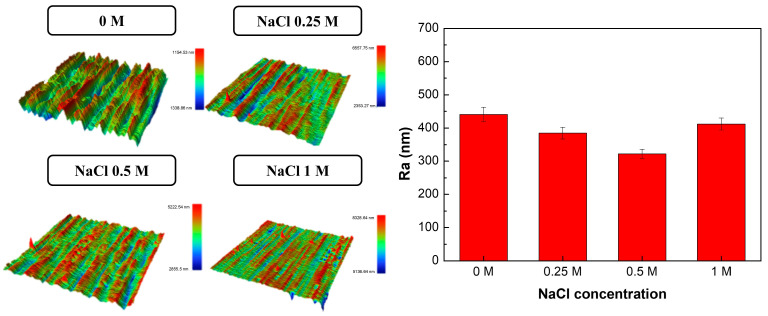
Surface morphologies of AA 6061 surface treatment, and R_a_ values with various NaCl concentrations.

The surface morphology was investigated by a profiler which is shown in Figure 3 and Table 1. A decrease in Ra was due to the removal of the oxide layer and pitting corrosion. However, Ra value decreased to 0.5 M and then increased again, but it could be observed that the Rp value representing the maximum cross-sectional peak height and the Rv value representing the maximum cross-sectional valley depth increased. These increased values explain that the increase in NaCl concentration leads to an increase in corrosion rates, resulting in numerous pits, and an increase in the roughness of the deviations. In the case of 1 M NaCl, the deepest craters occurred, which produced many rough irregularities and increased the R_a_, R_p_, and R_v_ values. This surface morphology can be explained using the above-mentioned Cassie–Baxter model, and which explains the increase in water contact angle.

**Figure 4:**
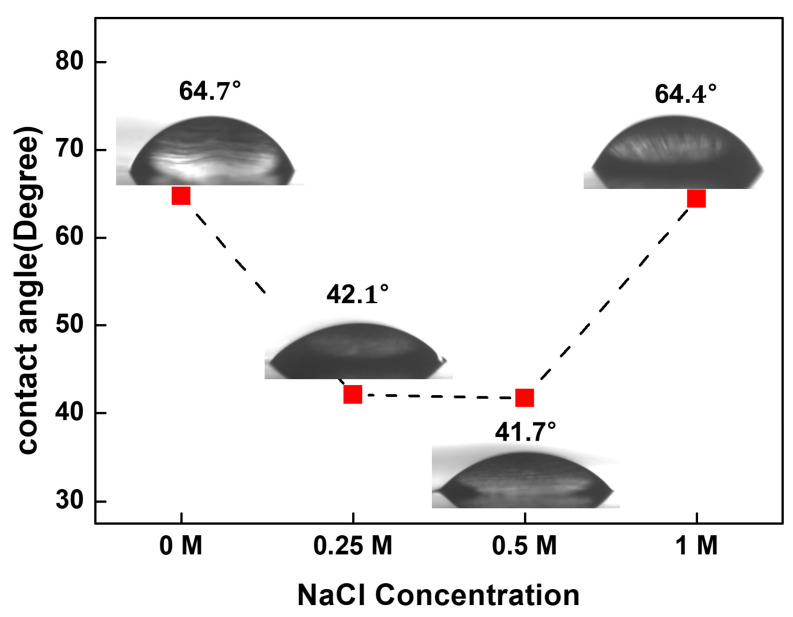
Contact angle of DI water on AA 6061 with various concentrations of NaCl after surface treatment at 50 ℃ for 1 h.

Figure 4 shows that the wettability of surfaces treated under different conditions was characterized using the water contact angle measurements. The measurements showed that the water contact angle decreased to approximately 40° on 0.25 M and 0.5 M NaCl on treated surfaces compared to 64.7° on the untreated surface. The contact angle of the 1 M NaCl-treated surface contact angle increased to 64.4°. Metals have a high surface energy and a hydrophilic surface. For the hydrophilic surface, increased roughness leads to a lower contact angle and the surface transforms to hydrophobic. The result of increased surface roughness of AA 6061 is an increased contact angle. Several investigations have shown that the surface of AA 6061 becomes more hydrophilic because of the functional group of hydroxides. According to the chemical composition of aluminum oxide in aqueous solutions, the main reaction that occurs between the –OH groups resulted from the etching process. Furthermore, the hydrophilic carboxyl groups from ascorbic acid lead to the formation of R–COO-Al. The long-chain alkyl group of ascorbic acid formed due to chemical bonding with the AA 6061, and caused a chemical reaction on the surface, which changed the surface energy. However, the contact angle decreased up to 0.5 M NaCl and then increased again. As described above, it can be confirmed that the reason for the decrease is that the surface of 6061 becomes more hydrophilic because of its reaction to ascorbic acid, which leads to a decreased water contact angle of 41.7°. The increase in the water contact angle at 1.0 M NaCl is explained by the Wenzel model and the Cassie–Baxter model [13]. The Wenzel equation, which is $\cos\theta_{w}=r\cdot \cos\theta_{\gamma }$, where *r* is the roughness factor of the ratio between the areas of rough and smooth surfaces. The Cassie–Baxter model explains the heterogeneous wettability, where air is trapped within the surface pores beneath the water droplet. The interface between solid and water decreased while the interface between the air and water increased, and then the water droplets on the surface become spherical. The Cassie–Baxter equation is as follows $\cos\theta_{c}=f\cos\theta_{\gamma }+f-1$, where *f* is the ratio of the solid surface in contact with the liquid. According to the Cassie–Baxter equation, a decrease in the *f* value means that air is easily trapped, and then the roughness and the contact angle increase. Both the Wenzel and Cassie–Baxter models explain that the contact angle increases with increasing roughness. Therefore, at 1.0 M NaCl, water drops do not penetrate the rough irregularities of the surface, because the rough irregularities are filled with air [14,15,16,17,18,19,20,21]. Thus, increased roughness prevented water drops from penetrating the surface, which led to an increase in water contact angle for 1 M NaCl. These findings corroborate previous reports by L. Feng et al. that hydroxide functional groups on the AA 6061 surface enhance hydrophilicity [22]. In addition, since the alkyl group is hydrophobic, the alkyl group of R–COO-Al formed by the reaction with ascorbic acid also affects the increase in the contact angle.

**Figure 5:**
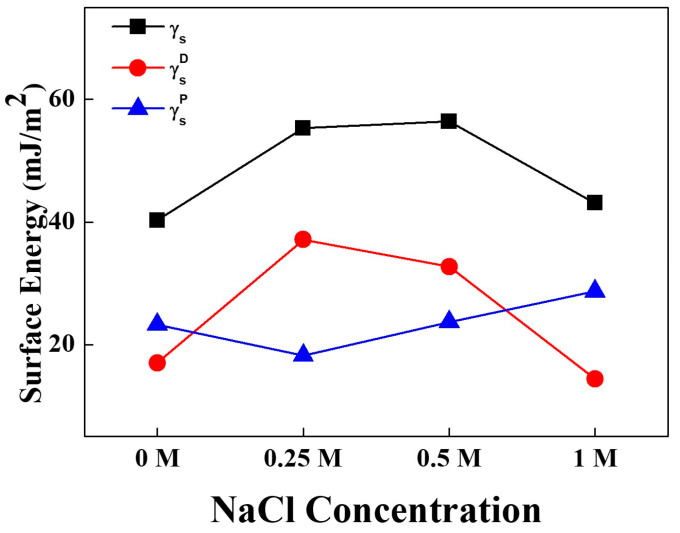
Calculated surface energies after surface treatment.

The OWRK method was used to calculate the surface energy [23,24,25,26]. The OWRK equation is as follows:

(1) $$\cos\theta_{\gamma_{lv}}=\gamma_{sv}-\gamma_{sl}-\pi_{e}$$

(2) $$\cos\theta_{\gamma_{lv}}=\left(1\right)\gamma_{c}=\gamma_{sv}-\gamma_{sl}-\pi_{e}$$

(3) $$\gamma_{s}=\gamma_{s}^{D}+\gamma_{s}^{P}$$

where $\gamma_{s}^{D}$ is dispersion energy and $\gamma_{s}^{P}$ is polar energy. The dispersion energy initially increased from 16.99 to 37.11 mJ/m^2^ and then decreased to 14.43 mJ/m^2^. Concurrently, the polar energy exhibited values of 23.27, 18.17, 23.69, and 28.67 mJ/m^2^, respectively. The total surface energy calculated using the OWRK equation yielded values of 40.26, 55.28, 56.41, and 43.1 mJ/m^2^, respectively. The surface free energy increased up to 0.5 M NaCl, and the highest surface free energy value was obtained at 0.5 M NaCl. When the concentration of NaCl was above 0.5 M, surface free energy decreased as shown in Figure 5 and Table 2.

**Figure 6:**
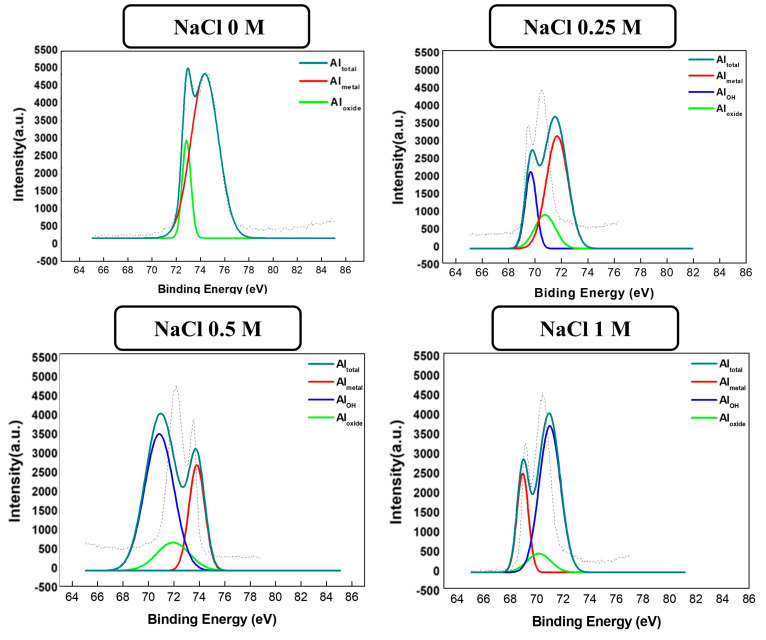
XPS spectra of Al surface treated with various concentrations of NaCl.

To investigate the changes in the surface composition of Al after the treatment with ascorbic acid and NaCl solutions, the treated surfaces were analyzed by XPS as illustrated in Figure 6 and Table 3. The surfaces exhibited three distinct peaks corresponding to Al metal (Al_met_, 72.8 eV), Al oxide (Al_Ox_, 74.1 eV), and Al hydroxide (Al_OH_, 74.8 eV) for all treatment conditions. [27,28,29]. The untreated surface exhibited two peaks, i.e., Al_met_ and Al_Ox_. Al_met_ showed a similar peak intensity for all conditions. In 1.0 M ascorbic acid, the Al metal and aluminum oxide ratio was 45.4% and 54.6%, respectively. When treated with a 0.25 M NaCl solution, Al metal and aluminum oxide value decreased to 21.5% and 17.6%. However, aluminum hydroxide was produced when treated with NaCl. The Al_Ox_ peak intensity decreased from 17.6% to 9.6% when the concentration of NaCl increased. This means that the oxide layer on the surface was further removed when the concentration of NaCl increased. The Al_OH_ peak tends to increase from 60.9% to 66.4% when the concentration of NaCl increases from 0.25 M to 1 M. When pitting corrosion occurs, the surface exposes Al ions, and exposed Al ions react with OH ions to form Al hydroxide in an aqueous solution [30]. Therefore, when the NaCl concentration increased, more pitting corrosion occurred, which promoted the formation of Al hydroxide on the surface.

**Figure 7:**
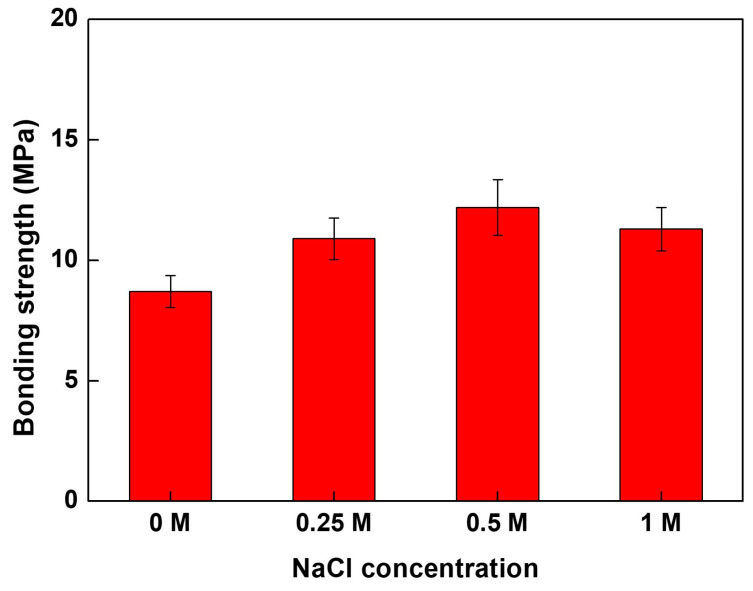
Bonding strength of AA 6061 and CFRP joints with various NaCl concentrations.

The bonding strength of the adhesive was evaluated by the single-lap joint shear test by ASTM D5868-01. Figure 7 illustrates the bonding strength results. The surfaces treated with 0, 0.25, 0.5, and 1 M NaCl exhibited bonding strengths of 8.7, 11.3, 13.8, and 12.1 MPa, respectively. This represents an increase of 58.6% for the 0.5 M treatment compared to the NaCl 0 M treatment. Bonding strength gradually increased until 0.5 M NaCl; however, bonding strength decreased above 0.5 M NaCl. The removal of an oxide layer and an increase in roughness provided higher bonding strength; however, excessive roughness and deep craters prevented the adhesive from penetrating into the Al surface, which decreased adhesion between Al and adhesive [13,14,15,16,17,18,19,20,21]. These bonding strength results show that the treatment concentration of 0.5 M NaCl was essential to achieving adequate roughness.

**Figure 8:**
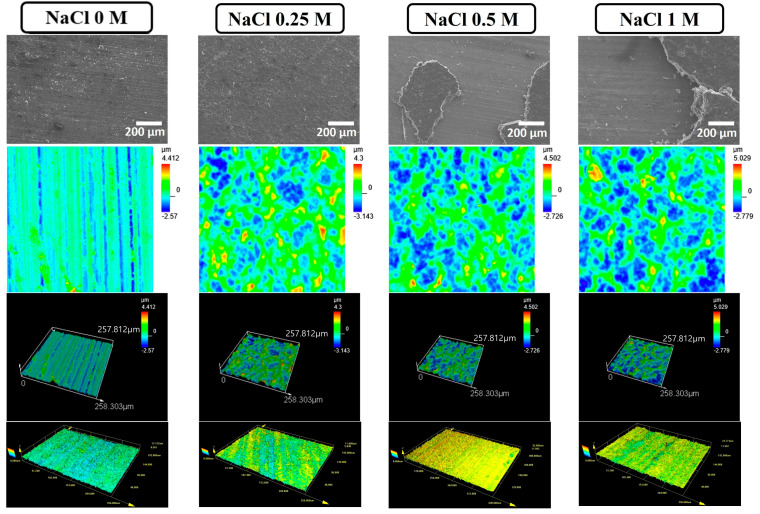
Cross-sectional micrographs and surface morphologies of AA 6061 fractured surfaces of various NaCl concentrations.

Figure 8 shows the fracture images of AA 6061 and CFRP. As a result of the fractured surface, it can be observed that in the case of NaCl 0 M, an interfacial fracture occurred on the AA 6061 side, and almost no adhesive remained on the surface of AA 6061. The reason for this is that, as confirmed through cross-sectional micrographs and surface morphology, in the case of NaCl 0 M, the surface roughness is lowest and only a small amount of pits were formed, so that the adhesive did not penetrate the Al metal. Although interfacial failure occurred in 0.25 M NaCl, it can be observed that a small amount of adhesive remained on the surface of AA 6061, which confirms that the mechanical interlocking was increased due to an increase in roughness. As the concentration of NaCl increased with an increase in residual adhesive and adhesive wettability on the Al surface, the bonding strength increased and changed from an interfacial failure to a cohesive failure.

**Figure 9:**
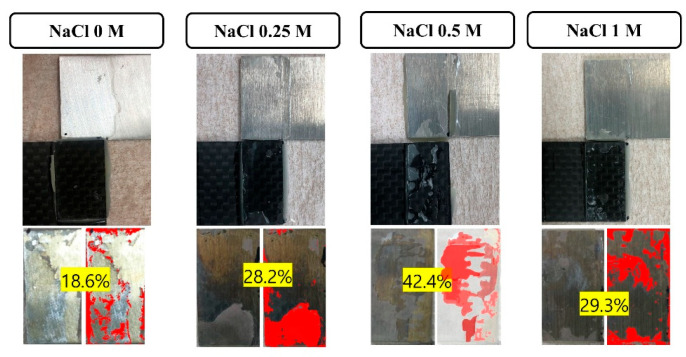
Fracture surface and residual adhesive surface ratio after lap shear test.

As shown in Figure 9, the highest cohesive fracture area (42.4%) was observed in the 0.5 M NaCl treatment. This demonstrates that, for certain adhesive systems, the macroscopic mechanical interlocking caused by deep craters induced by sodium chlorides improves bond strength better than surface free energy alone. Increased roughness allows the low-viscosity epoxy to penetrate deep into the AA 6061 surface, fixing the composite and shifting the stress field into the material during shear loading.

## 4. Conclusions

A simple and eco-friendly AA 6061 alloy surface etching process was used; specifically, the surfaces were treated with 1 M ascorbic acid solutions containing various concentrations of NaCl. A microstructural characterization revealed that an increase in NaCl concentration promoted pitting corrosion, generating numerous craters and increasing surface roughness. While the 0.25 M NaCl treatment showed a marginal effect, concentrations of 0.5 M and above effectively induced the desired pickling effect. However, excessive pitting corrosion at 1.0 M NaCl led to an increase in the water contact angle (64.4°) due to air entrapment within the surface irregularities. Furthermore, XPS analysis confirmed that the treated surfaces exhibited the formation of aluminum hydroxide (AlOH), which was not detected on the surface treated with NaCl 0 M. Increasing the NaCl concentration accelerated the removal of the passive aluminum oxide (AlOx) layer and exposed active Al ions that subsequently formed Al hydroxide.

These surface roughness and chemical composition influenced the mechanical properties of the adhesive joints. Single-lap shear tests demonstrated that the maximum bonding strength was achieved at 13.8 MPa at the 0.5 M NaCl condition, representing a 58.6% enhancement compared to the 0 M NaCl condition. Fracture surface analysis indicated that the increased surface roughness improved the wettability of the adhesive on the AA 6061 surface. Specifically, at the 0.5 M NaCl condition, the area of residual adhesive on the fractured surface increased to 42.4%, indicating a shift from interfacial fracture to cohesive failure attributed to enhanced mechanical interlocking. As a result, the ascorbic acid and NaCl offer a highly efficient etching process.

## Figures and Tables

**Table 1:** Values of Ra, Rp, and Rv with various NaCl concentrations.

	NaCl Concentration			
	0 M	0.25 M	0.5 M	1 M
R_a_ (nm)	441.4	384.6	322.4	412.1
R_p_ (nm)	1154.9	6597.8	5227.9	8328.6
R_v_ (nm)	−3338.9	−2352.3	−2895.5	−9136.6

**Table 2:** Calculated surface energy with various concentrations of NaCl.

		NaCl Concentration		
	0 M	0.25 M	0.5 M	1.0 M
$\gamma_{s}^{D}$ (mJ/m^2^)	16.99	37.11	32.72	14.43
$\gamma_{s}^{P}$ (mJ/m^2^)	23.27	18.17	23.69	28.67
$\gamma_{s}$ (mJ/m^2^)	40.26	55.28	56.41	43.1

**Table 3:** Relative concentration of the compounds obtained by XPS analysis with various concentrations of NaCl.

Al Type_tot_ (Total)		NaCl Concentration		
	0 M	0.25 M	0.5 M	1.0 M
Al (%)	45.4	21.5	25.3	24
Al_2_O_3_ (%)	54.6	17.6	14.0	9.6
Al(OH)_3_ (%)	-	60.9	60.7	66.4

## Data Availability

The original contributions presented in this study are included in the article. Further inquiries can be directed to the corresponding author.
